# A Two-Dimensional Scale for Oral Discomfort

**DOI:** 10.3390/ijerph22030415

**Published:** 2025-03-12

**Authors:** Yvonne A. B. Buunk-Werkhoven, Dick P. H. Barelds, Arie Dijkstra, Abraham P. Buunk

**Affiliations:** 1 Faculty of Medicine, Kauno Kolegija, LT-50468 Kaunas, Lithuania; 2 Organizational Psychology, Faculty of Behavioral and Social Sciences, University of Groningen, 9712 CP Groningen, The Netherlands; d.p.h.barelds@rug.nl; 3 Social Psychology, Faculty of Behavioral and Social Sciences, University of Groningen, 9712 CP Groningen, The Netherlands; arie.dijkstra@rug.nl (A.D.); a.p.buunk@rug.nl (A.P.B.)

**Keywords:** oral discomfort, psychological discomfort, physical discomfort, psychometrics, validation study

## Abstract

Subjective suffering due to oral diseases and disorders has been conceptualized as oral health-related quality of life and is often assessed with a multidimensional version of the Oral Health Impact Profile (OHIP). In the current study, a secondary analysis of a Dutch-language translated version of the original OHIP-14 was performed in different samples of approximately 1000 participants from diverse contexts (i.e., The Netherlands, the Caribbean, and Nepal). The dimensional structure and reliability of the scales resulting from these analyses were also examined. Based on a number of Confirmatory Factor Analyses (CFAs) and Simultaneous Components Analysis (SCA) of the OHIP-14 scale, testing various models with different numbers of factors, several models were acceptable, but a two-factor solution, comprising *psychological discomfort* and *physical discomfort* was the most satisfactory in all three samples, although a one-factor solution, *oral discomfort* was also acceptable. Instead of using a large number of dimensions with a few items each, as often is done, it is most adequate and feasible to use no more than two scales, i.e., *psychological discomfort* and *physical discomfort*, comprising 11 items in total. These subscales of six and, respectively, five items are not only statistically, but also theoretically, the most adequate. Additionally, all items together, i.e., *oral discomfort* as a one-dimensional scale, are useful and easy to apply for practical use.

## 1. Introduction

Quality of life (QoL) is a very important concept in health care, particularly for the development and evaluation of health interventions that intend to maintain or improve people’s health. Health-related QoL (HRQoL) can be considered the subjective perception of one’s personal position in life related to one’s health [1]. In general, it is a very important aspect of many medical problems and chronical disorders [2], which also applies to oral health conditions, such as tooth decay (caries). Caries can be considered a chronic disease and the most common prevalent disease in both children and adults, and, even though it is largely preventable, it may affect one’s quality of life [3].

For the development of adequate and tailored oral health interventions, it is a necessary prerequisite to accurately estimate the relative impact of chronic oral diseases on oral health-related quality of life (OH-QoL), so that health care resources can be better planned and allocated to achieve optimal OH-QoL [4]. Quality of life in relation to dental or oral health is a very subjective concept. Therefore, it is important to have a valid and reliable scale to assess OH-QoL. When a scale to measure a psychological attribute, for instance, OH-QoL is developed, the often-recommended procedure is to select a representative sample of items from the domain representing the attribute. This approach is called domain sampling theory [5]. In this sample of items, the true dimensionality of the target attribute can be identified. This implies that if other authors would like to develop another measure of the same attribute, the same dimensions would be found in a different set of items from the domain. It follows that if a set of items is selected that is not a domain sample and a dimensionality analysis is applied, the real dimensions of the target construct may not be identified.

The present research focuses on the dimensionality of a measure of OH-QoL that is considered here as subjective oral health. Based on the WHO framework for impairment, disability and handicap (1980) a quarter century ago, Kay and Locker [6] (p. 8) defined oral health as “a standard of health of the mouth and related tissues that enables a person to eat, speak, and socialize without active disease, discomfort or embarrassment and that contributes to general well-being”. Building upon this definition, currently oral health is specified and considered a “multifaceted phenomenon, including the ability to speak, smile, smell, taste, touch, chew and swallow, as well as the ability to express a range of emotions through facial expressions, with confidence and without pain, discomfort and disease of the craniofacial complex [6]”. In order to place more focus on the personal and self-experienced, it is assumed that subjective oral health is influenced by an individual’s perceptions, expectations and ability to adapt to different circumstances [7,8]. OH-QoL is an essential aspect of general health throughout life and essential for the overall quality of life (QoL) [9,10]. Especially, psychosocial well-being is an important aspect of patients’ OH-QoL [11,12].

The goal of the present research is to develop a single measure or two measures of OH-QoL that are easy to use and interpret in practice and in research. In 2010, based on previous studies [13,14], Buunk-Werkhoven proposed interpreting the OH-QoL concept as ‘dental satisfaction’ and suggested that the Oral Health Impact Profile-14: OHIP-14 measures a single construct to determine the degree of an individual’s own perceived dental satisfaction. Indeed, in one study, an exploratory factor analysis (EFA) showed one appropriate factor for the used dataset [15,16]. Hence, to develop reliable and valid scales for OH-QoL, the present study examines the structure of the items of the widely used OHIP-14 [17,18].

Although the original OHIP-14 is based on the OHIP, a 49-item instrument that can be considered a domain sample of the construct OH-QoL, it has—from a psychometric perspective—a number of limitations. First, it is not a set of items representative of the domain of OH-QoL. Although in the study by Slade [18], the OHIP-14 accounted for 94% of the variance in the OHIP-49, that does not mean that all dimensions are represented [19]. Second, its dimensional nature consists of seven scales, each of two items. Such scales are not very reliable; according to psychometric insights, reliability depends on the length of the scale and the correlations between the items. Calculating a Cronbach’s alpha, which is commonly used as a measure of a test’s reliability [20] for two items, does not make much sense and is in fact not really possible.

A third limitation is that the reported distribution of the items is generally very skewed, and any restriction of the range may have had a negative effect on both reliability and validity. A fourth limitation is that with its seven dimensions, the OHIP-14 is not very useful in research that wants to predict the OH-QoL from other variables, such as personality, age, dental visits, or attitude towards oral health care. Although review studies recommend a four-dimensional framework to cover the different domains of OH-QoL, i.e., psychosocial impact (formerly *handicap*), oral function, orofacial pain, and orofacial appearance [12,21,22,23], for practical application, it is also better to be able to work with one or two scales, for example, to evaluate patients’ reported OH-QoL, for screening large groups of patients or for interpreting population-level trends and for purposes like clinical trials and performance measurement. Also, in medical research, only a few scales are usually used to measure similar constructs. However, when developing an individual treatment plan, profile scores based on multiple dimensions can provide specific insight and would be useful for a personalized intervention focused on that one problem area.

Finally, it is important to clarify that the OHIP-14 does not directly measure OH-QoL. Instead, it more accurately reflects *oral discomfort*, which aligns better with the content of the items. By contrast, and given the psychological and experiential aspects of quality of life, *oral discomfort* is influenced not only by objective dental health status, but also by factors like oral hygiene behavior, knowledge, lifestyle, gender, occupational status and cultural influences [19,24,25]. Thus, with the goal of developing one or two scales to measure *oral discomfort* that are content-valid, structured and easily to apply in practice, the present research examines the psychometric structure of the translated and culturally adapted Dutch version of the short OHIP-14 [26], which has been carefully translated backward-forward [27]. With the aim of further improving the quality of this measurement instrument, the translation was performed by two different professional translators working independently, and the final version was refined, modified and validated in several population-based studies [28,29,30,31,32,33]. In addition, more specific insight in factors associated with mouth-related, e.g., *oral discomfort* or oral health issues, and self-care practices is of great importance for developing tailored oral health care interventions in the daily practice of oral health professionals [4].

## 2. Method

### 2.1. Ethical Statement and Procedure

Permission for all studies was obtained from the ethical committee of the Faculty of Behavioral and Social Sciences, University of Groningen. In the period of June 2023 to April 2024, this secondary analysis of data from a number of other studies was conducted according to universal ethical principles and in line with the Helsinki Declaration. Participation was completely free, and anonymity was guaranteed. It was made clear to the participants that they were free to stop at any moment filling out the questionnaire. As many measures as possible were taken to control patient selection by making participating in the research as easy as possible, by using a brief questionnaire, by having a set-up that prevented dropping out of the study (which indeed rarely occurred) and by using the window of opportunity (for example, patients who had to wait before treatment).

### 2.2. Sample Description

The *Dutch sample* included 790 participants/patients (45% female), with a mean age of 28.8 (*SD* = 18.8). Twenty-two percent of the Dutch participants had a low level of education, 23% had a medium level, and 54% had a high level of education. In this total sample, 555 participants were students and military recruits of about the same average age [28,31]. The remaining participants were divided into 112 patients who attended a dental school [13], 80 forensic psychiatric male patients [28,30] and 43 elderly people [33].

The *Caribbean sample* included 113 participants/patients (55% female, with a mean (SD) age of 36.5 (13.2) years, who visited a dental practice in Bonaire and in Aruba. Although Dutch is the official language, Papiamento—a mixture of Portuguese, Spanish, English and Dutch words—as the native language is spoken by 73% as its mother tongue. In the Caribbean sample, 48% of the participants were married, 5% had a low level of education, 74% had a medium level and 23% had a high level of education [34].

The *Nepalese sample* included 108 participants (54% female), with a mean (SD) age of 40.1 (16.5) years, who visited a dental camp of The Netherlands Oral Health Society (NOHS) in the region of Newalparasi. The Dutch OHIP-14 version was translated into Sanskrit and was filled out by 69 participants, whereas the data for 39 participants were collected through a semi-structured interview by a Nepalese translator. The Nepalese translator was an official female translator and a medical professional in midwifery and preventive health care. This sample is a multi-ethnic group of people related to Brahmin, Magar, and Newari, Tharu, Chetri and Gurung backgrounds. Nepali as the national language is spoken by 90% as their mother tongue. Seventy-four percent of the participants in the sample were married, and the level of education varied from no education (28%), low (27%), and medium (32%) to a high level (13%) [34]. Because of the three different nationalities in the sample and to verify that the OHIP-14 was face- and content-validated for each of these nationalities, the procedure of Geisinger [35] was partially used in the backward-forward translation of the items from Dutch into the national mother tongue. Substantial care was taken with respect to the language issue. A multiply culturally adapted paper-and-pencil-questionnaire was checked again by a native speaker/researcher-professional, and moreover people on the Dutch Antilles are fully bilingual [34]. Discrepancies were reviewed several times, and any differences and concerns were reconciled with the translation until it was agreed that the language was clear and understandable for the sample population and that the OHIP-14 tapped the intended construct in this sample.

To be completely sure, each participant was asked if he or she understood the items [34].

### 2.3. Measures

*Oral health-related quality of life*. To assess OH-QoL, all items of the original OHIP-14 were used. The original version [9] is designed so that questions on a given topic are presented sequentially. The fact that these questions appear together may encourage similar responses to each, regardless of the actual meaning of the questions. In other words, the answers chosen may be an artefact of the position of a question [29,32]. Therefore, we shuffled the order of the items to encourage respondents to answer each question independently. This instrument consists of 14 items organized—the original order was shuffled in the administration of the scale—in seven dimensions each of two items, i.e., *function limitation* (‘speaking’ + ‘sense of taste’), *physical pain* (‘painful aching’ + ‘uncomfortable eating’), *psychological discomfort* (‘self-conscious’ + ‘tension’), *physical disability* (‘unsatisfactory diet’ + ‘interrupt meals’), *psychological disability* (‘difficult to relax’ + ‘embarrassed’), *social disability* (‘irritable’ + ‘occupational’) and *handicap* (‘unsatisfactory life’ + ‘unable to function’). Responses were scored on a 5-point Likert scale (i.e., 0 = ‘never’, 1 = ‘sometimes’, 2 = ‘regularly’, 3 = ‘often’ and 4 = ‘very often’). Please note, hence, that the original answer options in the OHIP-14 were slightly adapted, i.e., 1 = ‘hardly never’ was changed into ‘sometimes’ and 2 = ‘occasionally’ was changed into ‘regularly’. From a psychometric perspective, these two original responses’ wordings often showed unequal distance in discrimination, generally causing a skewed distribution. In Dutch psychological research, ‘never’, ‘sometimes’, ‘regularly’, ‘often’ and ‘very often’ are commonly used response options for distance discrimination. Even a possible translation issue such as changing from ‘occasionally’ to ‘regularly’ could potentially affect the variance. However, equal discrimination in response alternatives may better reflect the distribution of the prevalence of impact responses. Moreover, zero was defined as the maximal positive result indicative of total absence of problems and 4 corresponded to a maximal negative answer or always a problem. A total general OHIP-14 score for each respondent was calculated as the sum of all the 14 items.

Noteworthily, Guttman’s lambda 2 coefficient was used here as an alternative measure of reliability. Removing items from OHIP scales may affect the estimate of reliability and is justified if there is a rationale for it and face validity is still maintained [20]. In addition, decisions regarding the omission of items were made against the background of extensive qualitative findings (from interviews and observations [28,30,31]) on the meaning that people attach to the applied formulations (Data available upon request).

### 2.4. Statistical Analyses

A description of the psychometric properties evaluated or statistical tests conducted follows: First, it was decided, rather arbitrarily, to start by examining solutions with one, two or three components. Of these three, the solution with three components was uninterpretable. Therefore, it was decided not to examine solutions with more than three components. The solution with two components was conceptually the best interpretable solution and was also supported by the Scree plot.

Second, Confirmatory Factor Analyses were used to evaluate the overall model fit of four potential factor models that have previously been proposed in the literature (models with 7, 3, 3, and 1 factors, respectively) in the three samples. Lisrel [36] was used for these CFAs. In addition, Simultaneous Components Analyses (SCAs) were conducted, a technique for *exploratively* examining a factor structure across multiple samples [37]. SCA is specifically designed to identify consistent patterns across different datasets without imposing strict model constraints beforehand (like in a CFA). This is useful when there is still uncertainty about how the items behave across different samples [38,39]. The SCAs were conducted using the Matlab program (version 9.13.0; R2022b) [40]. As a next step, it was evaluated to what extent the factors found in the SCAs (that is across samples) resembled the factors found per sample by means of an Exploratory Factor Analysis (EFA). This way, it was possible to see to what extent the most optimal structure per sample (the EFA solution) resembled the exploratory structure across samples (the SCA structure). To examine this, congruence coefficients were computed between the factors from the final SCA structure and the EFA structures, again using Matlab [40]. In addition, by means of additional CFAs (in Lisrel), the overall model fit per sample of the structure found in the SCA was checked. The reliability of the resulting final components was examined by calculating Gutmann’s λ2, which is a lower bound to the reliability, just like Cronbach’s alpha, but a better estimate of the reliability [20]. These values were calculated in SPSS (version 27) [41]. Mean differences between samples were finally examined by means of a MANOVA, also conducted in SPSS.

## 3. Results

First, the structure of the original OHIP-14 was examined by means of Confirmatory Factor Analyses (CFAs). Based on previous studies on the structure of the OHIP-14, four potential factor models (described as A to D below) were tested in the present study’s three subsamples. The results of the CFAs are summarized in Table 1. For the meaning of the various Q indices, see Table 2.

- Seven-factor solution, which represents the original seven factors identified by Locker [42]: function limitation (Q4, Q14), physical pain (Q2, Q5), *psychological discomfort* (Q1, Q9), physical disability (Q12, Q10), psychological disability (Q13, Q8), social disability (Q7, Q3) and handicap (Q11, Q6). These factors were also used in developing the OHIP-14 [16,18].
- Three-factor solution that was found in a Dutch sample using the OHIP-14 [27]: functional limitations (Q2, Q3, Q4, Q5, Q10, Q14), social discomfort (Q7, Q8, Q9, Q11, Q12, Q13) and psychological inhibitions (Q1, Q6).
- Three-factor solution, as reported in a Spanish sample [17] and as further examined in Brazilian samples [15]: psychosocial impact (Q1, Q9, Q13, Q8, Q7, Q3, Q11, Q14), pain-discomfort (Q2, Q5, Q12, Q10) and functional limitations (Q4, Q14).
- One-factor solution: original (14 items) and final *oral discomfort* scale (11 items).

In order to assess the model fit, the chi-square test, the root mean square error of approximation (RMSEA), the comparative fit index (CFI) and the Tucker–Lewis index (TLI) were used (see Table 1), and a cut-off value close to or less than 0.06 for RMSEA and a cut-off value close to or greater than 0.95 for CFI and TLI as indicators of acceptable model fit were recommended [43].

As can be seen from Table 1, there is little difference between the fit indices of the four models in the three samples. Generally speaking, the original seven-factor model (Model A) fits a little bit better than the two three-factor models (Models B and C) and the one-factor model (Model D). The RMSEA values are not satisfactory, regardless of model or sample though (all values are 0.087 or higher), and the TLI values are consistently below 0.95 for the Caribbean and Nepalese samples. Based on these outcomes, there appears not to be a clearly superior model fitting the data in these three samples. The RMSEA values at or above the levels reported in Table 1 and Table 3 are not unusual for the results of CFAs applied to patient-reported outcome data, due to the skewed nature of such data [44].

It was therefore decided to exploratively examine the structure of the original OHIP-14. The joint dataset was used to find the structure that best fits all three samples. More specifically, Simultaneous Components Analysis (SCA) was used, which is particularly suitable for finding the common structure across different datasets that share the same variables. The differences in variability across the various taxonomies were removed, so that the joint analysis was done on the common correlation structure. There are different variants of SCA [45,46], of which the SCA-ECP version was used here (this is a SCA with equal cross-products). Solutions with one, two and three components were examined. It turned out that the three-factor solution was uninterpretable. A solution with two factors, however, was conceptually the best interpretable. The rotated solution (oblimin solution) is reported in Table 2.

The first component can be interpreted as *psychological discomfort* and the second component as *physical discomfort*. To examine the extent to which this SCA-ECP solution fit the factor structure in each of the three samples independently, congruencies between the two SCA-ECP components are reported in Table 2, and the explorative two-component solutions (principal axis factoring with oblimin rotation) for each sample were calculated. The congruencies (Tucker’s ɸ) were calculated. A ɸ of 0.85 to 0.94 indicates fair similarity, while values of 0.95 or higher imply that two components can be considered equal [47]. It turned out that in all samples, the congruencies were good for the first component, i.e., *psychological discomfort*, for the Dutch sample ɸ = 0.99, for the Caribbean sample ɸ = 0.97 and for the Nepal sample ɸ = 0.96. The congruency was also good for the second component, i.e., *physical discomfort*, in the Dutch sample, ɸ = 0.98, and in the other samples the congruencies indicated only fair similarity for the Caribbean sample ɸ = 0.94, and for Nepalese sample ɸ = 0.87.

Based on these outcomes, it was decided to retain this two-component solution and only retain those items with an absolute factor loading of 0.40 or higher. This means that items Q4, Q6 and Q14 were dropped from further analyses (see Table 2). All three items did not load strongly on one of both presumed factors (all < 0.40). In addition, all three were less easy to classify as referring to the psychological or physical oral domain. To examine the overall model fit of this two-factor solution, based on 11 items, CFAs were conducted on the three subsamples (see Table 3). For comparison purposes, two models were tested: one with the two factors as reported in Table 2 and a one-factor solution (‘*oral discomfort*’; Model D in Table 1). The two models showed a comparable fit in the Dutch and Nepalese samples, with slightly better values of the fit indices for the two-factor solution. In the Caribbean sample, the two factors clearly outperformed the one-factor solution.

Next, in all three samples for the remaining 11 items, the reliability of the two components *psychological discomfort* and *physical discomfort* was estimated. For this purpose, Guttman’s lambda 2 coefficient was calculated, which is preferable to Cronbach’s alpha [20]. The λ2 reliabilities in the Dutch and Caribbean samples were good (for *psychological discomfort* 0.98 and 0.94, respectively and for *physical discomfort* 0.87 and 0.86. respectively). The λ2s were sufficient in the Nepalese sample (0.73 for *psychological discomfort* and 0.76 for *physical* discomfort). On the basis of the factor analyses, two scales are proposed for *oral discomfort*, and the final versions of these scales are presented in Table 4.

Group differences between the samples were examined by means of a multivariate analysis of variance. There was a significant multivariate effect of sample, *F*(4, 2014) = 17.07, *p* < 0.01, as well as significant univariate effects for both *psychological discomfort*, *F*(2, 1008) = 31.07, *p* < 0.01 and *physical discomfort*, *F*(2, 1008) = 20.37, *p* < 0.01. Pairwise comparisons showed that all means differed significantly between the samples (*p*’s < 0.01), with the exception of the means for *physical discomfort* of the Dutch and the Caribbean samples (*p* = 0.96). For *psychological* and *physical discomfort*, the means were for The Netherlands *M =* 0.36 (*SD* = 0.58) and *M* = 0.48 (*SD* = 0.61), respectively, for the Caribbean sample *M =* 0.58 *SD =* 0.74) and 0.54 (*SD* = 0.64), respectively, and for the Nepalese sample, *M =* 0.85 *SD =* 0.86) and 0.90 (*SD* = 0.87), respectively. These results show that the Dutch sample reported the lowest discomfort scores and the Nepalese sample the highest.

## 4. Discussion

The purpose of the present study was to examine the dimensional structure and the reliability of a Dutch version of the Oral Health Impact Profile-14, with the aim of developing one or two scales to measure *oral discomfort* that are valid in terms of content, structured and easy to apply in practice. Initially, like previous studies [15,48], Confirmatory Factor Analysis (CFA) was used. In addition, Simultaneous Components Analysis (SCA) was used, which is particularly suitable for finding the common structure across different datasets that share the same variables [37,38,39]. The results of this study provide evidence of how this OHIP-14 instrument works in different samples.

Based on this, a two-dimensional scale, with dimensions labelled as *psychological discomfort* and *physical discomfort*, seems to best fit the data, and seems, moreover, an adequate measurement tool for clinical and research purposes. The two dimensions are theoretically very sensible and are more appropriate than the seven dimensions of the OHIP-14 for use in research. Moreover, the two scales can easily be used and interpreted in clinical practice, giving practitioners a direct sense of the psychological and physical oral health of their patients. In the two-factor solution, it was found that three items of the original OHIP-14 did not correlate consistently with the remaining items, and they were dropped. Two deleted items in the used OHIP-14, i.e., ‘speaking’ and ‘sense of taste’, formed in the original OHIP-14 [42] one of the seven dimensions, namely *function limitation*. The item ‘unable to function’ was also removed from the scale presented here, though in the original OHIP scale, this particular item OHIP-14, combined with OHIP-13 (‘unsatisfactory life’), formed the dimension *handicap*. It may be noted that also in previous studies, the item ‘unable to function’ correlated inconsistently with the other items. However, in this study there was overlap and only some shifts of items compared to the three subscales described in a previous study by Buunk and Buunk-Werkhoven [28]; for example, here, *psychological discomfort* is interpreted as compared to ‘social discomfort’ and *physical discomfort* as compared to ‘functional limitations’ respectively.

To summarize, the present research suggests that what is often referred to as oral health-related quality of life can be best conceptualized as consisting of two dimensions measured by two brief scales that are easy to administer. The *psychological discomfort* subscale encompasses the affective aspects of *oral discomfort*, including tension, dissatisfaction and embarrassment related to oral health conditions or treatments. The *physical discomfort* subscale evaluates sensory experiences such as pain and eating problems directly associated with teeth, mouth, or dentures. In addition, there is also psychometric support for a one-factor solution of overall *oral discomfort* that can be measured with the final 11-item scale, i.e., two subscales together, which can be useful and easy to apply for practical use (see Table 4). If the length of the questionnaire (i.e., the time needed for its completion by participants or patients) and interpretation, or, for instance, the number of dimensions are an issue [16,21,49,50], the scales presented here may be optimal, and practitioners as well as researchers can choose to look only at the scores of the total 11-item version or to examine the scores of the two subscales. Especially in various situations, for example, when one wants to evaluate the effect of a treatment or when one is interested in examining the level of *oral discomfort* in a specific patient or population [49,51,52], the final 11-item scale can be a useful tool that does not require too much time of patients and will therefore result in low non-response levels.

The present research has a number of limitations. The first limitation of this study is that most data came from quite specific populations, and, thus, in the future, the scales should be administered to samples representative of the population, also to obtain norm scores for the scales, so that users can assess to what extent the score of a patient or group is high or low. A second potential limitation is that the data from three different samples were pooled, and, given somewhat different translation methods and potential mode effects, the data may not have been completely comparable. However, all care was taken to make the questionnaires similar across countries and therefore eligible to be pooled. Moreover, cross-cultural research is very important for the advancement of scientific knowledge, and it is unavoidable that data cannot always be completely similar in different cultures. Notably, the oral health data from Caribbean and Nepalese samples are quite unique, but an unknown selection may have taken place which may underly the slight differences in scale quality between these and the Dutch sample. Future studies on these cultures will have to further improve the sampling methods.

A third limitation is that there were no data on the validity of the scales that could be assessed, for example, by relating the scales to similar instruments. A fourth potential limitation is that it has yet not been demonstrated how useful the scales are in practice. Nevertheless, the scales included only items from the original OHIP-14 that have been widely used. A final potential limitation is that although the order of the original OHIP-14 questions was shuffled to encourage participants to independently consider different questions on the same topic, the fact that the OHIP-14 was administered in a nonstandard order may have compromised the comparability of the results measured with the *oral discomfort* scale with those based on data collected using the original format. Future research should determine whether the *oral discomfort* outcomes are replicated in the analysis of data derived from the OHIP-14 as originally formatted.

Overall, quantitative research on the outcomes of oral disorders, as perceived and reported by patients and individuals, is required to further innovate dental and oral care. Therefore, a universally applicable *oral discomfort* measure to ensure consistency of assessing and measuring outcomes that more explicitly address issues and discomfort related to the mouth should be adopted [17,18,19,26,28,32]. The scales proposed in the present research might play a role in future applied research to further study its characteristics, for example, the convergent and discriminant validity of this *oral discomfort* scale.

In practice, the scale might be used to develop interventions aimed at improving oral quality of life and its related oral health behaviour. This means appropriately applying the Problem-Analysis-Test-Help-Success (PATHS) model, i.e., different phases must be distinguished for designing effective interventions [4]. This means that in the P-phase, the potential target population should be defined. In this phase, the present *oral discomfort* scales might help to identify groups, subgroups and individuals with the most urgent needs. In the following A- and T-phases, a broad set of determinants of *oral discomfort* should be assessed and analysed. For example, the *oral discomfort* scale score might be related to dental care organization, treatment practices and individual behaviours. In the H-phase, the actual intervention package is developed to improve *oral comfort* by targeting population-specific determinants [31]. *Oral discomfort* might be an integral part of the intervention procedure as a screening instrument. Lastly, in the S-phase, the effectiveness of the intervention must be assessed, for example, with the present *oral discomfort* scale. When the intervention is effective, the intervention may be widely implemented in practice.

## 5. Conclusions

The present research suggests that an adapted version of the original OHIP-14, consisting of two scales, i.e., *psychological discomfort* and *physical discomfort*, may be the most adequate approach to measuring what is usually labelled as oral health-related quality of life, but that may be better referred to as *oral discomfort*. The two subscales together can be easily used in practice and research to assess patients’ *oral discomfort* in terms of *psychological* and *physical discomfort* at the intake, as well at the end of treatment, to examine the effect of a treatment on subjective oral health.

## Figures and Tables

**Table 1:** Results from the CFAs of three samples.

	**The Netherlands (*n* = 790)**			
	*X* ^2^	RMSEA (90% CI)	CFI	TLI
Model A	415.92	0.087 (0.079–0.095)	0.98	0.97
Model B	619.18	0.098 (0.091–0.11)	0.97	0.96
Model C	610.88	0.097 (0.090–0.10)	0.97	0.96
Model D	730.21	0.11 (0.10–0.11)	0.96	0.96
	**Caribbean (*n* = 113)**			
	*X* ^2^	RMSEA (90% CI)	CFI	TLI
Model A	140.18	0.11 (0.088–0.14)	0.96	0.94
Model B	193.53	0.12 (0.095–0.14)	0.95	0.93
Model C	186.48	0.12 (0.095–0.14)	0.95	0.94
Model D	269.88	0.18 (0.16–0.20)	0.91	0.90
	**Nepal (*n* = 108)**			
	*X* ^2^	RMSEA (90% CI)	CFI	TLI
Model A	121.85	0.098 (0.071–0.12)	0.93	0.89
Model B	149.65	0.093 (0.070–0.12)	0.92	0.90
Model C	155.63	0.094 (0.071–0.12)	0.91	0.89
Model D	157.61	0.092 (0.069–0.11)	0.91	0.90

**Table 2:** Rotated SCA-ECP solution.

Original OHIP-14 with Shuffled Item Order	*Psychological Discomfort*	*Physical Discomfort*
Q1 = ‘self-conscious’	**0.465**	0.122
Q2 = ‘painful aching’	0.048	**0.580**
Q3 = ‘occupational’	0.294	**0.544**
*Q4 = ‘speaking’*	0.397	0.234
Q5 = ‘uncomfortable eating’	−0.086	**0.895**
*Q6 = ‘unable to function’*	0.377	0.247
Q7 = ‘irritable’	**0.756**	−0.010
Q8 = ‘embarrassed’	**0.728**	−0.013
Q9 = ‘tension’	**0.739**	0.087
Q10 = ‘interrupt meals’	0.302	**0.470**
Q11 = ‘unsatisfactory life’	**0.939**	−0.176
Q12 = ‘unsatisfactory diet’	0.390	**0.453**
Q13 = ‘difficult to relax’	**0.719**	0.076
*Q14 = ‘sense of taste’*	0.362	0.274

Note: selected items per component are printed in **bold**; the not selected items are printed in *italic*.

**Table 3:** Results from the CFAs in three samples based on 11 items.

	**The Netherlands (*n* = 790)**			
	*X* ^2^	RMSEA (90% CI)	CFI	TLI
Two factors	398.63	0.11 (0.096–0.11)	0.97	0.96
One factor	586.84	0.13 (0.13–0.14)	0.96	0.94
	**Caribbean (*n* = 113)**			
	*X* ^2^	RMSEA (90% CI)	CFI	TLI
Two factors	70.35	0.073 (0.039–0.10)	0.98	0.98
One factor	183.59	0.20 (0.18–0.25)	0.91	0.89
	**Nepal (*n* = 108)**			
	*X* ^2^	RMSEA (90% CI)	CFI	TLI
Two factors	92.22	0.091 (0.060–0.12)	0.92	0.90
One factor	95.18	0.094 (0.064–0.12)	0.92	0.90

**Table 4:** The final *oral discomfort* measure as a two-dimensional scale (11 items) [*psychological discomfort* (1, 2, 3, 4, 5, 6) and *physical discomfort* (7, 8, 9, 10, 11)].

*Oral Discomfort*	0	1	2	3	4
1. Have you been self-conscious because of your teeth, mouth, or dentures?	never	sometimes	regularly	often	very often
2. Have you been a bit irritable with other people because of problems with your teeth, mouth, or dentures?	never	sometimes	regularly	often	very often
3. Have you been a bit embarrassed because of problems with your teeth, mouth, or dentures?	never	sometimes	regularly	often	very often
4. Have you felt tense because of problems with your teeth, mouth, or dentures?	never	sometimes	regularly	often	very often
5. Have you felt that life in general was less satisfying because of problems with your teeth, mouth, or dentures?	never	sometimes	regularly	often	very often
6. Have you found it difficult to relax because of problems with your teeth, mouth, or dentures?	never	sometimes	regularly	often	very often
7. Have you had painful aching in your mouth?	never	sometimes	regularly	often	very often
8. Have you had difficulty doing your usual jobs because of problems with your teeth, mouth, or dentures?	never	sometimes	regularly	often	very often
9. Have you found it uncomfortable to eat any foods because of problems with your teeth, mouth, or dentures?	never	sometimes	regularly	often	very often
10. Have you had to interrupt meals because of problems with your teeth, mouth, or dentures?	never	sometimes	regularly	often	very often
11. Has your diet been unsatisfactory because of problems with your teeth, mouth or dentures?	never	sometimes	regularly	often	very often

Note: The Dutch, Nepalese and Indonesian versions of the final *Oral Discomfort* Scale can be obtained from the first author.

## Data Availability

The dataset for this study is in a repository and is available at the Open Science Framework (*OSF*; https://osf.io/wuyfr/ accessed 7 March 2024). The DOI is as follows: https://doi.org/10.17605/OSF.IO/WUYFR.

## References

[1] Haraldstad K., Wahl A., Andenæs R., Andersen J.R., Andersen M.H., Beisland E., Borge C.R., Engebretsen E., Eisemann M., Halvorsrud L. (2019). LIVSFORSK network. A systematic review of quality of life research in medicine and health sciences. Qual. Life Res..

[2] Megari K. (2013). Quality of Life in Chronic Disease Patients. Health Psychol. Res..

[3] Giacaman R.A., Fernández C.E., Muñoz-Sandoval C., León S., García-Manríquez N., Echeverría C., Valdés S., Castro R.J., Gambetta-Tessini K. (2022). Understanding dental caries as a non-communicable and behavioral disease: Management implications. Front. Oral. Health.

[4] Buunk A.P., Dijkstra P., Van Vugt M. (2021). Applying Social Psychology.

[5] Crocker L., Algina J. (1986). Introduction to Classical and Modern Test Theory.

[6] Kay E., Locker D. (1997). Effectiveness of Oral Health Promotion: A Review.

[7] Buunk-Werkhoven Y.A.B., Dijkstra A., van der Schans C.P. (2011). Determinants of oral hygiene behavior: A study based on the theory of planned behavior. Community Dent. Oral. Epidemiol..

[8] Glick M., Williams D.M., Kleinman D.V., Vujicic M., Watt R.G., Weyant R.J. (2016). A new definition for oral health developed by the FDI World Dental Federation opens the door to a universal definition of oral health. J. Am. Dent. Assoc..

[9] Locker D. (2004). Oral health and quality of life. Oral. Health Prev. Dent..

[10] Myers-Wright N., Lamster I.B. (2016). A New Practice Approach for Oral Health Professionals. J. Evid. Based Dent. Pract..

[11] Bennadi D., Reddy C.V. (2013). Oral health related quality of life. J. Int. Soc. Prev. Community Dent..

[12] Su N., van Wijk A., Visscher C.M. (2021). Psychosocial oral health-related quality of life impact: A systematic review. J. Oral. Rehabil..

[13] Buunk-Werkhoven Y.A.B., Dijkstra A., van der Schans C.P. (2009). Oral health-quality of life predictors depend on population. Appl. Res. Qual. Life..

[14] Buunk-Werkhoven Y.A.B. (2010). World White Teeth: Determinants and Promotion of Oral Hygiene Behavior in Diverse Contexts. Ph.D. Thesis, Thesis Fully Internal (DIV).

[15] Santos C.M., Oliveira B.H., Nadanovsky P., Hilgert J.B., Celeste R.K., Hugo F.N. (2013). The Oral Health Impact Profile-14: A unidimensional scale?. Cad. Saude Publica.

[16] Silveira M.F., Marôco J.P., Freire R.S., Martins A.M., Marcopito L.F. (2014). Impact of oral health on physical and psychosocial dimensions: An analysis using structural equation modeling. Cad. Saude Publica.

[17] Montero J., López J.F., Vicente M.P., Galindo M.P., Albaladejo A., Bravo M. (2011). Comparative validity of the OIDP and OHIP-14 in describing the impact of oral health on quality of life in a cross-sectional study performed in Spanish adults. Med. Oral. Patol. Oral. Cir. Bucal..

[18] Slade G.D. (1997). Derivation and validation of a short-form oral health impact profile. Community Dent. Oral. Epidemiol..

[19] Locker D., Allen F. (2007). What do measures of ‘oral health-related quality of life’ measure?. Community Dent. Oral. Epidemiol..

[20] Sijtsma K. (2009). On the use, the misuse, and the very limited usefulness of Cronbach’s alpha. Psychometrika.

[21] Campos L.A., Peltomäki T., Marôco J., Campos J.A.D.B. (2021). Use of Oral Health Impact Profile-14 (OHIP-14) in Different Contexts. What Is Being Measured?. Int. J. Environ. Res. Public. Health.

[22] Ingleshwar A., John M.T. (2023). Cross-cultural adaptations of the oral health impact profile—An assessment of global availability of 4-dimensional oral health impact characterization. J. Evid. Based Dent. Pract..

[23] John M.T., Omara M., Su N., List T., Sekulic S., Häggman-Henrikson B., Visscher C.M., Bekes K., Reissmann D.R., Baba K. (2022). Recommendations for Use and Scoring of Oral Health Impact Profile Versions. J. Evid. Based Dent. Pract..

[24] Baker S.R. (2007). Testing a conceptual model of oral health: A structural equation modeling approach. J. Dent. Res..

[25] Sakki T.K., Knuuttila M.L., Anttila S.S. (1998). Lifestyle, gender and occupational status as determinants of dental health behavior. J. Clin. Periodontol..

[26] Werkhoven Y.A.B., Spreen M., Buunk A.P., Schaub R.M.H. (2004). Mondzorg in de Dr. S. van Mesdagkliniek heeft meer om het lijf. [Oral health care in Dr. S. van Mesdag Forensic Psychiatric Centre: More than oral health care alone]. GGzet Wetenschappelijk.

[27] Buunk-Werkhoven Y.A.B., Verheggen-Udding E.L., van den Heuvel J.L. (2011). Mondgezondheidgerelateerde levenkwalteit bij patiënten met terbeschikkingstelling [Oral health related quality of life among Dutch forensic psychiatric patients]. Ned. Tijdschr. Tandheelkd..

[28] Buunk A.P., Buunk-Werkhoven Y.A.B. (2018). Sense of defeat, Social status and Oral health among forensic psychiatric patients. Eur. J. Med. Nat. Sci..

[29] Buunk-Werkhoven Y.A.B., Dijkstra A., van der Wal H., Basic N., Loomans S.A., van der Schans C.P., van der Meer R. (2009). Promoting oral hygiene behavior in recruits in the Dutch Army. Mil. Med..

[30] Buunk-Werkhoven Y.A.B., Dijkstra A., Schaub R.M., van der Schans C.P., Spreen M. (2010). Oral health related quality of life among imprisoned Dutch forensic psychiatric patients. J. Forensic. Nurs..

[31] Buunk-Werkhoven Y., Dijkstra-le Clercq M., Verheggen-Udding E., de Jong N., Spreen M. (2012). Halitosis and oral health-related quality of life: A case report. Int. J. Dent. Hyg..

[32] Buunk-Werkhoven Y.A.B., van den Heuvel H.B. Dutch recruits’ and students’ oral health-related quality of life. Proceedings of the Abstracts of the 100th FDI Annual World Dental Congress.

[33] Rijpstra X. (2007). Oral Health of Elderly in a Nursing Home in Relation to Quality of Life. [Mondgezondheid van ouderen in een verzorgingshuis in relatie tot Kwaliteit van Leven]. Master’s Thesis.

[34] Buunk-Werkhoven Y.A.B., Dijkstra A., Bink P., van Zanten S., van der Schans C.P. (2011). Determinants and promotion of oral hygiene behaviour in the Caribbean and Nepal. Int. Dent. J..

[35] Geisinger K.F. (1994). Cross-cultural normative assessment: Translation and adaptation issues influencing the normative interpretation of assessment instruments. Psychol. Assess..

[36] Jöreskog K.G., Sörbom D. (1993). LISREL 8: Structural Equation Modeling with the SIMPLIS Command Language.

[37] Kiers H.A.L., ten Berge J.M.F. (1994). Hierarchical relations between methods for simultaneous component analysis and a technique for rotation to a simple simultaneous structure. Br. J. Math. Stat. Psychol..

[38] De Raad B., Barelds D.P.H., Timmerman M.E., De Roover K., Mlačić B., Church A.T. (2014). Towards A Pan–Cultural Personality Structure: Input from 11 Psycholexical Studies. Eur. J. Pers..

[39] Timmerman M.E., Kiers H.A.L., Ceulemans E. (2016). Searching components with simple structure in simultaneous component analysis: Blockwise Simplimax rotation. Chemometr Intell. Lab. Syst..

[40] The MathWorks Inc. (2022). MATLAB.

[41] IBM Corp (2020). IBM SPSS Statistics for Windows.

[42] Locker D. (1988). Measuring oral health: A conceptual framework. Community Dent. Health.

[43] Hu L.-T., Bentler P.M., Hoyle R.H. (1995). Evaluating model fit. Structural Equation Modeling: Concepts, Issues, and Applications.

[44] Cook K.F., Kallen M.A., Amtmann D. (2009). Having a fit: Impact of number of items and distribution of data on traditional criteria for assessing IRT’s unidimensionality assumption. Qual. Life Res..

[45] De Roover K., Ceulemans E., Timmerman M.E. (2012). How to perform multiblock component analysis in practice. Behav. Res. Methods.

[46] Timmerman M.E., Kiers H.A.L. (2003). Four simultaneous component models for the analysis of multivariate time series from more than one subject to model intraindividual and interindividual differences. Psychometrika.

[47] Lorenzo-Seva U., Ten Berge J.M.F. (2006). Tucker’s congruence coefficient as a meaningful index of factor similarity. Methodology.

[48] John M.T., Reissmann D.R., Feuerstahler L., Waller N., Baba K., Larsson P., Celebić A., Szabo G., Rener-Sitar K. (2014). Exploratory factor analysis of the Oral Health Impact Profile. J. Oral. Rehabil..

[49] Mulders G., van Verseveld H., van der Geer J., Wolvius E., Leebeek F. (2023). The state of oral health in patients with haemophilia in the Netherlands. Haemophilia.

[50] van der Meulen M.J., John M.T., Naeije M., Lobbezoo F. (2012). Developing abbreviated OHIP versions for use with TMD patients. J. Oral. Rehabil..

[51] Gasparro R., Di Spirito F., Cangiano M., De Benedictis A., Sammartino P., Sammartino G., Bochicchio V., Maldonato N.M., Scandurra C. (2023). A Cross-Sectional Study on Cognitive Vulnerability Patterns in Dental Anxiety: The Italian Validation of the Dental Fear Maintenance Questionnaire (DFMQ). Int. J. Environ. Res. Public. Health.

[52] Tan M.L., Tuk J.G., Markarian V., de Lange J., Lindeboom J.A. (2023). Assessing change in quality of life using the Oral Health Impact Profile in patients undergoing orthognathic surgery: A before and after comparison with a minimal follow-up of two years. J. Stomatol. Oral. Maxillofac. Surg..

